# Downward Repositioning of Breast Mound with Early Phase Intervention for Autologous Breast Reconstruction Patients

**DOI:** 10.1055/a-2525-5772

**Published:** 2025-03-24

**Authors:** Makoto Shimabukuro, Naohiro Ishii, Naohiko Ikura, Kyoichi Matsuzaki, Kazuo Kishi

**Affiliations:** 1 Department of Plastic and Reconstructive Surgery, International University of Health and Welfare Hospital, Tochigi, Japan; 2 Department of Plastic and Reconstructive Surgery, International University of Health and Welfare, School of Medicine, Chiba, Japan; 3 Department of Plastic and Reconstructive Surgery, Tochigi Cancer Center, Tochigi, Japan; 4 Department of Plastic and Reconstructive Surgery, Keio University, Tokyo, Japan

**Keywords:** breast mound, ptosis, reconstruction, skin flap, sponge

## Abstract

In breast reconstruction with a flap transfer, symmetry is often difficult to achieve when the contralateral breast projection has a downward peak. Although minimally invasive and effective methods for postoperative correction of the reconstructed breast mound are desirable, none has been comprehensively reported. We devised a correction method comprising downward movement of the reconstructed breast mound using early postoperative dissection and pressure. This method was applied to four patients undergoing primary two-stage ptotic breast reconstruction with a flap transfer. All of their reconstructed breast mounds were positioned above the contralateral side in the early postoperative period. They underwent manual dissection of the upper edge in flaps under local anesthesia 3 weeks after reconstruction or downward pressure correction using a sponge for 6 months or both procedures. The reconstructed breast mound moved 2 to 2.5 cm downward with early postoperative manual dissection or pressure correction using a sponge and moved 3 cm downward with the combination of both dissection and pressure. Manual dissection in the early postoperative period under local anesthesia and compression with a sponge is minimally invasive and useful for the downward correction of the reconstructed breast mound. The combination of dissection and compression provides a greater corrective effect.

## Introduction

Breast reconstruction using autologous tissue after mastectomy is widely performed, and breast symmetry is important for patient satisfaction. However, it is often difficult to reproduce the symmetry of ptotic breasts owing to the insufficient volume of the skin flap, the need to fix the skin flap on the cephalic side because of the vascular anastomotic position, and inaccurate evaluation of the bilateral ptotic morphology because of intraoperative posture problems.

To reproduce ptotic breasts, the skin flap volume has been increased or decreased, and healthy breasts have been modified 1 2 ; however, these methods are not minimally invasive. In Japan, revision surgery for healthy breasts is not often performed owing to the psychological resistance to inserting a scalpel in healthy tissue; additionally, it is not covered by insurance.

We developed and applied a novel method comprising early postoperative upper edge dissection under local anesthesia and sponge compression for the downward correction of the reconstructed breast mound after the primary two-stage reconstruction of ptotic breasts.

## Idea

We applied a method of downward correction comprising early postoperative upper margin dissection under local anesthesia and sponge compression for the reconstructed breast mound of four patients from 2017 to 2020. Primary two-stage ptotic breast reconstruction was performed with an abdominal free flap for three patients, with a pedicle latissimus dorsi flap for one patient. The anastomotic position (internal thoracic artery and vein) in the reconstruction with an abdominal free flap was positioned cranially so that the reconstructed breast mound was positioned above the contralateral side in all cases. The pedicle latissimus dorsi flap was misplaced, causing the reconstructed breast mound to be positioned above the contralateral side.

**Fig. 1:**
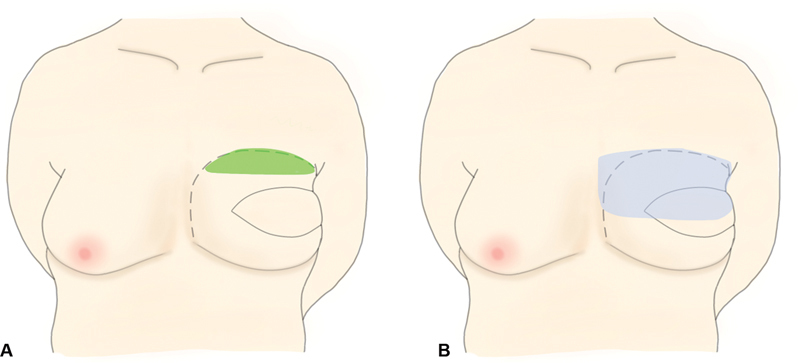
A comprehensive explanation and illustration showing the surgical procedure of early flap dissection and compression. ( **A** ) We removed the fixation threads from the upper border of the flap by approaching the upper skin suture line under local anesthesia and performed dissection around the fixed skin flap. Refixation of the flap was not performed. The green area shows the area of early flap dissection, and the dotted line shows the margin of the transferred flap. ( **B** ) A sponge (Reston pad®; 3M, St. Paul, MN) is placed on the upper pole of the reconstructed breast mound, and a breast band is placed over the sponge to compress and fix the reconstructed breast mound downward. The blue area shows the area of compression using the sponge, and the dotted line shows the margin of the transferred flap.

For two patients, we performed a revision procedure with upper margin dissection under local anesthesia 3 weeks postoperatively ( Fig. 1A ). For three patients, sponge compression was applied from the head of the reconstructed breast ( Fig. 1B ). For one patient, both procedures were applied. All patients had worn a corrective brassiere for a year after flap transfer. We measured the downward movement of the reconstructed breast mound with respect to its inferior margin with four patients treated with this method. The xiphoid process was the reference point for the measurements.

**Fig. 2:**
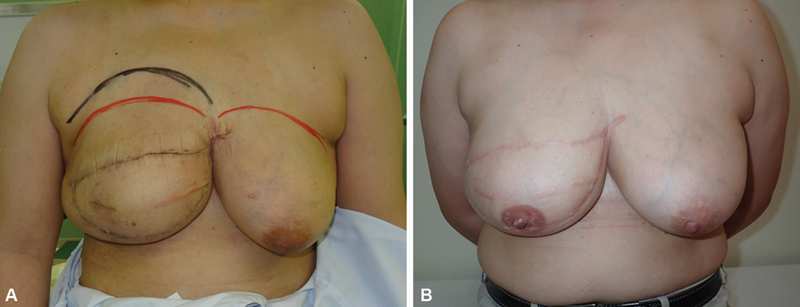
Progression of case 1. ( **A** ) At 2.5 weeks postoperatively, the height of the upper margin of the reconstructed breast and peak protrusion of the reconstructed breast are higher than those of the healthy side. ( **B** ) Photograph after upper margin dissection and 1.5 years postoperatively.

Patient characteristics and the results of all present cases are shown in Table 1 . No complications were observed postoperatively. The reconstructed breast mounds spontaneously and gradually dropped, and just right downward shifts were achieved; consequently, all cosmetic results were much improved ( Figs. 2 3 4 5 ). Furthermore, we also measured postoperative caudal skin flap migration 2 years postoperatively with respect to the inferior margin of the reconstructed breast in randomly selected cases involving transverse rectus abdominis musculocutaneous flaps combined with tissue expander that were not indicated for our downward correction method. For these cases, only corrective undergarments that held the entire reconstructed breast in place were used for 6 months. The results are shown in Table 2 . We compared the present four cases with these cases, and patients treated with this method had more downward displacement of the flap than did patients treated without this method.

**Table 1:** Patient characteristics and results of downward movement of reconstructed breast mound in cases in which our method was performed

	Breast size a	Regnault classification	Body mass index (kg/m ^2^ )	Kinds of flap used	Start date of upper margin dissection of the flap (A) or pressure correction (B)	Period of postoperative pressure correction with a sponge	Distance difference immediately after flap transfer (cm)	Distance difference immediately after upper margin dissection of the flap (cm)	Distance difference 2 years postoperatively
Case 1	Large	Grade 3	25.8	DIEP/TRAM	A: 3 weeks after flap transfer	–	2.5	1	0 cm
Case 2	Large	Grade 1	29.2	DIEP	B: 1 month after flap transfer	5 months	2	–	0 cm
Case 3	Medium	Grade 2	23.7	TRAM	A: 3 weeks after flap transfer B: 1 month after flap transfer	5 months	3	1.5	0 cm
Case 4	Medium	Grade 1	26.0	LD	B: 3 weeks after flap transfer	3 months and 1 week	2	–	−0.5 cm

Abbreviations: DIEP; deep inferior epigastric perforator flap; LD; latissimus dorsi musculocutaneous flap; TRAM; transverse rectus abdominis musculocutaneous flap.

Distance difference: distance difference between the lower edge of the breast mound of the reconstructed side and that of the healthy side (+, the former is higher than the latter, −, the former is lower than the latter).

a Small, A or B cup; medium, C or D cup; large, E cup or larger.

**Fig. 3:**
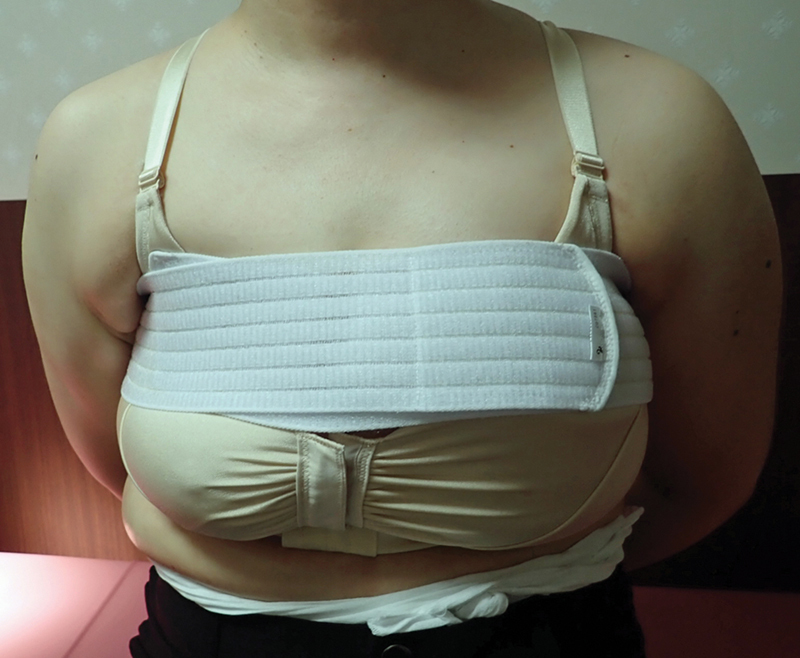
A photo of case 2. Photograph of downward pressure correction using a sponge, which was started 1 month postoperatively.

**Fig. 4:**
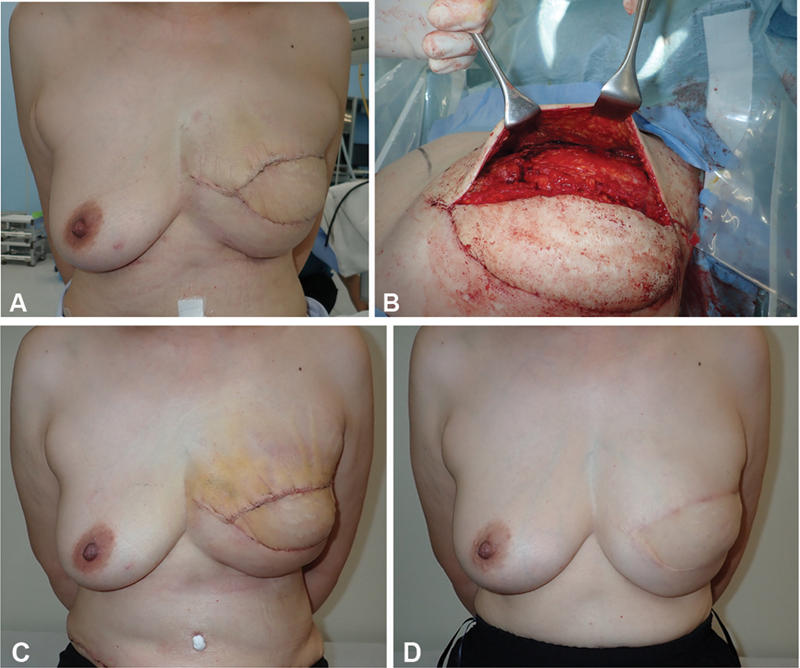
Progression of case 3. ( **A** ) At 3 weeks postoperatively, the height of the upper margin of the reconstructed breast and peak protrusion of the reconstructed breast are higher than those of the healthy side. ( **B** ) The patient underwent upper margin dissection at 3 weeks postoperatively. ( **C** ) Photograph obtained 1 week after upper margin dissection. ( **D** ) Photograph after upper margin dissection and subsequent pressure correction using a sponge at 2 years postoperatively.

**Fig. 5:**
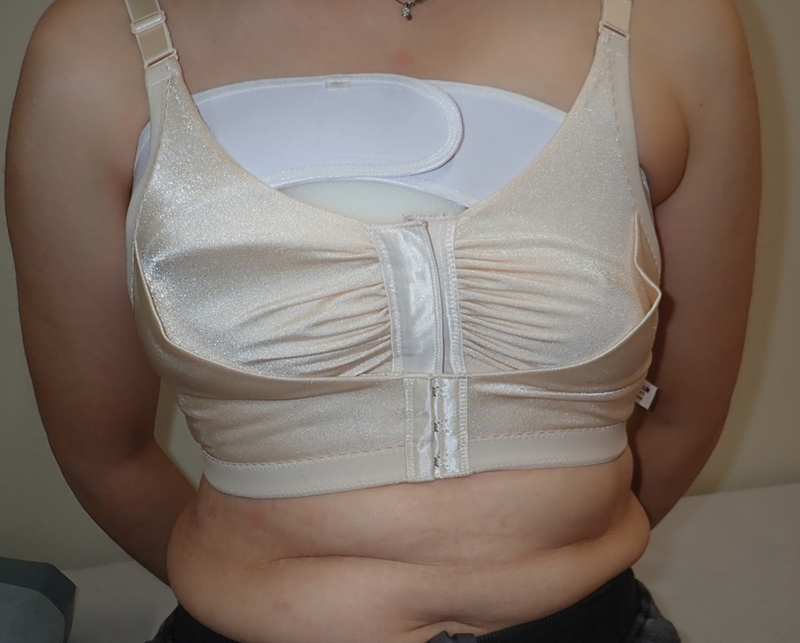
A photo of case 4. This photograph was obtained when downward pressure correction using a sponge was started 1 month postoperatively.

**Table 2:** Downward movement of reconstructed breast mound in cases in which our method was not performed

	Breast size a	Grade of Regnault classification, means (range)	Body mass index (kg/m ^2^ ), means (range)	Distance difference immediately after flap transfer, means (range)	Distance difference 2 years postoperatively, means (range; cm)
Comparative group 1, *N* = 10	Small	0.0 (0–0)	20.3 (18.1–22.3)	0.7 (0.2–0.9) cm	−0.2 (−0.8 to 0.4)
Comparative group 2, *N* = 10	Medium	0.8 (0–2)	23.8 (22.0–25.4)	0.9 (0.4–1.1) cm	−0.2 (−0.7 to 0.3)
Comparative group 3, *N* = 10	Large	1.9 (1–3)	25.6 (23.1–30.3)	1.1 (0.7–1.3) cm	−0.3 (−0.6 to 0.3)

Distance difference: distance difference between the lower edge of the breast mound of the reconstructed side and that of the healthy side (+, the former is higher than the latter; −, the former is lower than the latter).

a Small, A or B cup; medium, C or D cup; large, E cup or larger.

Written informed consent was obtained from all patients for the publication of this article and accompanying images.

## Discussion

The initial corrective procedure that is commonly performed to achieve stronger expression of the ptotic morphology of the reconstructed breast mound comprises the reduction of cephalic fat and the addition of caudal fat of the reconstructed breast. The former involves liposuction or direct visual fat removal, 3 whereas the latter involves fat injection grafting. Additionally, when a large increase in volume, including that of the skin, is required, another skin flap, such as the latissimus dorsi flap, intercostal artery perforator flap, or anterolateral thigh flap, may be implanted. 4 In contrast, mastopexy or reduction mammaplasty may be performed to reduce the degree of ptosis of the healthy breast, 1 and these methods may be performed in combination with the aforementioned reconstructive breast surgery. These procedures are necessary if the volume of the reconstructed breast mound is deficient compared with that of the healthy breast. However, if the total volume of the reconstructed breast mound is approximately equal to that of the healthy breast volume, then these procedures may be invasive; nevertheless, no minimally invasive correction method has been reported.

For cases 1 and 3, we performed a revision procedure with upper margin dissection under local anesthesia 3 weeks postoperatively, when the flap blood flow was stable, and the flap adhesion to pectoralis major muscle seemed relatively mild. We believe that removing the upper border threads and flap dissection of upper pole facilitates caudal migration of the flap to create a drooping effect. This operative technique is minimally invasive, easy, and effective. No similar procedure has been reported.

For cases 2, 3, and 4, at 1 month postoperatively, sponge compression with a breast band was applied in the upper pole of the reconstructed breast until 4 to 6 months postoperatively. Compression for flap repositioning of breast mound after breast reconstruction by autologous tissue transfer may be clinically applied; however, there have been no high-quality reports with detailed progress notes of this method. Compression therapy using a sponge is often used to reduce the elevation of keloids and hypertrophic scars. 5 In the present cases, the sponge was used to apply continuous pressure to the cephalic side of the reconstructed breast mound to allow gradual caudal migration. The sponge, which was thick, slightly firmer, and slightly elastic, was considered more effective for sustained pressure. One advantage of sponges is that they are inexpensive and can be used repeatedly. Certainly, this technique is also minimally invasive, easy, and effective, similar to our revision procedure for upper margin dissection.

A corrective brassiere that is designed to hold the breast in place is often used to prevent abnormal downward and lateral deviations of the reconstructed breast. Its effectiveness and importance have been advocated in Japan. 6 In the present cases, we suppose it contributed to preventing the downward migration of the inframammary fold (IMF). Additionally, a breast band may be worn around the upper edge of the breast to prevent upward deviation of the reconstructed breast mound during expander insertion or for breasts reconstructed with implants. In the present cases, a breast band was used for compression and fixation of the sponge. The breast band is an excellent fixation device that is inexpensive (approximately $15), can be used repeatedly, and has a fixation force that can be easily adjusted with hook-and-loop tape to apply pressure to the sponge without shifting.

A downward shift of approximately 1.5 cm was achieved immediately after revision surgery under local anesthesia. However, a downward shift of approximately 2 cm was achieved by sponge compression alone, and a downward shift of approximately 3 cm was achieved by the combination of dissection and compression. No complications, such as skin flap damage, were observed. We reviewed previous cases of breast reconstruction followed by the use of corrective brassieres alone and found that the average downward migration distance was 1 cm in the small breasts group, 1.1 cm in the medium breasts group, and 1.4 cm in the large breasts group ( Table 2 ). The results suggested that this method contributed to more downward displacement of the flap. This method may be applied appropriately when the lower edge of the reconstructed breast mound is 2 cm higher than that of the contralateral breast mound, the total flap volume is approximately equal to the volume of the contralateral breast, and the height of the upper margin of the flap and most prominent part of the skin flap is located higher than the contralateral side. A revision procedure with upper margin dissection may be applied when the lower edge of the reconstructed breast mound is 2.5 cm higher than that of the contralateral breast mound. Additionally, when applying this method, we recommend the reconstructed breast mound should be firmly supported by corrective brassiere to prevent an IMF left–right difference and the duration of sponge compression over the upper pole of the reconstructed breast should be adjusted flexibly according to morphological changes. Since our method was employed only in four cases, a larger case series is required to confirm these results, and the appropriate timing of the upper margin dissection of the flap should be considered. Furthermore, individual differences regarding the detailed surgical procedure, skin elasticity, flap weight, and flap atrophy or in the rate of thoracic skin expansion may have affected postoperative results, and this should be considered.

In conclusion, we performed early postoperative flap dissection and sponge compression under local anesthesia to move the reconstructed breast mound downward in four patients with ptotic breasts who underwent primary two-stage reconstruction with flap transfer. Consequently, an average downward shift of 2.5 cm (with regard to the inferior margin of the breast) of the breast mound was achieved.

Although it is often difficult to correct an upwardly positioned reconstructed breast mound, this useful, minimally invasive, convenient, and inexpensive technique resulted in satisfactory outcomes. Furthermore, we believe that the combination of early postoperative upper edge dissection and sponge compression provides a greater corrective effect.
